# Clinical efficacy and prognostic factors of CT-guided ^125^I brachytherapy for the palliative treatment of retroperitoneal metastatic lymph nodes

**DOI:** 10.1186/s40644-020-00299-x

**Published:** 2020-04-06

**Authors:** Huzheng Yan, Ma Luo, Lifei Wang, Zhenkang Qiu, Zhiqiang Mo, Zhanwang Xiang, Yanling Zhang, Guanyu Chen, Zhihui Zhong, Xiuchen Wang, Fei Gao, Fujun Zhang

**Affiliations:** 1 Sun Yat-sen University Cancer Center, 651 Dongfeng Road, East, Guangzhou, 510060 China; 2 State Key Laboratory of Oncology in South China, 651 Dongfeng Road, East, Guangzhou, 510060 China; 3 Collaborative Innovation Center for Cancer Medicine, 651 Dongfeng Road, East, Guangzhou, 510060 China; 4 The Department of Radiology, The Third People’s Hospital of Shenzhen, Shenzhen, China; 5 Department of Interventional Radiology, Guangdong General Hospital, Guangdong Academy of Medical Sciences, 106 Zhongshan No. 2 Road, Guangzhou, 510060 China; 6 The Third affiliated hospital, Sun Yat-sen University, Guangzhou, China

**Keywords:** ^125^I brachytherapy, ^125^I seed, Retroperitoneal metastatic lymph nodes

## Abstract

**Background:**

Due to the unique anatomical location of retroperitoneal metastatic lymph nodes, current treatment options are limited. This study was designed to explore the clinical efficacy and prognostic factors of CT-guided ^125^I brachytherapy for the treatment of retroperitoneal metastatic lymph nodes.

**Methods:**

We retrospectively evaluated 92 patients received ^125^I brachytherapy for retroperitoneal metastatic lymph nodes. A layered Cox proportional hazards model was established to filter out the independent factors affecting local tumor progression-free survival (LTPFS).

**Results:**

The median LTPFS was 8 months. Metastatic lymph node with uniform density (p-0.009), clear boundaries (p-0.011), regular morphology (*P* < 0.001), and < 3 organs at risk of metastasis (p-0.020) were associated with better LTPFS. Necrotic lymph nodes (*p* < 0.001), fusion (p-0.003), and invasion of vessels visible on images (*p* < 0.001) were associated with poor LTPFS. Puncture path through abdominal wall or paravertebral approach were also associated with better LTPFS than a hepatic approach (*P* < 0.05). A maximum diameter ≤ 3 cm (P-0.031) or 3–5 cm (P-0.018) were also associated with significantly better LTPFS than a maximum diameter ≥ 5 cm. The Cox proportional hazards model suggested that lymph nodes invaded the large vessels visible on images, maximum diameter and puncture path were independent risk factors for LTPFS.

**Conclusion:**

CT-guided ^125^I brachytherapy is an optional palliative treatment modality for retroperitoneal metastatic lymph nodes, which can provide high local control without severe complications. Better preoperative planning, intraoperative implementation, better choice of puncture path, and selection of appropriate tumor size are important factors that can improve the clinical efficacy of ^125^I brachytherapy for retroperitoneal metastatic lymph nodes.

## Background

Malignant tumors in the abdominal cavity, lower limbs, and other parts of the body can metastasize to the retroperitoneum via the lymphatic circulation [1]. Therefore, the retroperitoneal lymph nodes are the most common sites of metastasis for cancers, such as esophageal, gastric, hepatic, pancreatic, colorectal, ovarian, and cervical cancers. The retroperitoneum is adjacent to vital organs and structures, such as the pancreas, duodenum, ureter, large blood vessels, and nerves [2, 3]. Retroperitoneal metastatic lymph nodes often cause a series of serious clinical symptoms, such as abdominal pain, bloating, jaundice, loss of appetite, and radiating pain in the lower back. These symptoms can severely affect the patients’ quality of life [4].

Since the location of the retroperitoneal lymph nodes is deep and concealed, conventional surgical resection is difficult [5, 6]. It is difficult to maintain high concentrations of chemotherapies in the pelvic and abdominal lymph nodes, and systemic chemotherapy has little or no treatment effect [7–10]. Peritoneal hyperthermic perfusion has been used to concentrate drugs locally, and this can augment the antitumor response in retroperitoneal metastatic lymph nodes [11]. However, it is difficult to obtain better results for relatively large retroperitoneal metastatic lymph nodes. In recent years, new radiotherapy techniques, such as three-dimensional conformal intensity-modulated radiotherapy and stereotactic radiotherapy, have been shown to significantly reduce the dose of radiotherapy outside the target area. However, retroperitoneal metastatic lymph nodes are often close to the spinal cord, gastrointestinal tract, liver, kidney, and pancreas, which have extremely low radiation tolerance. Therefore, it is often difficult to decipher the risk/benefit ratio of radiation therapy [12–14].

In recent years, a new treatment, namely, ^125^I brachytherapy has emerged, in which radiolabeled ^25^I seeds are implanted into solid tumors through surgery or by image guidance. Low-dose radiation from the radioactive seeds then continuously emit β or γ-rays to kill or inhibit the growth of tumor cells. One of the advantages of brachytherapy is that radiation exposure is localized, thereby preventing off-target radiation damage to adjacent normal tissues [15]. As a result, brachytherapy is often used in patients with tumors in complex locations, or in patients who cannot tolerate traditional radiotherapy [16–18]. Due to their complex anatomical location, retroperitoneal metastatic lymph nodes are more suitable for this minimally invasive and safe ^125^I brachytherapy. Previously few studies have applied ^125^I brachytherapy for retroperitoneal metastatic lymph nodes [19, 20]. Therefore, this study was designed to explore the clinical efficacy and prognostic factors of CT-guided ^125^I brachytherapy for the treatment of retroperitoneal metastatic lymph nodes.

## Method

This retrospective study was approved by the Institutional Review Board of Sun Yat-sen University Cancer Center. We conducted a retrospective analysis of 92 patients with retroperitoneal metastatic lymph nodes, who were treated with ^125^I brachytherapy from April 2008 to August 2016.

### Inclusion and exclusion criteria

Inclusion criteria: (1) metastatic retroperitoneal lymph nodes; (2) the number of metastatic lymph node ≤5; (3) Age 18–70; and (4) ECOG score ≤ 2.

Exclusion criteria: (1) lack of key information required for research, such as CT, MRI, PET, and other imaging examinations, before and after treatment; (2) primary retroperitoneal malignant tumors; (3) extensive retroperitoneal metastatic lymph nodes; and (4) treatment with microwave ablation, radiofrequency ablation, and chemical ablation while receiving ^125^I brachytherapy.

### ^125^I brachytherapy

^125^I seeds (Yunke Pharmaceuticals Limited Liability Company, Chengdu, China) consists of a titanium tube with an outer diameter of 0.8 mm, a length of 4.5 mm and a wall thickness of 0.05 mm. The ^125^I isotope is attached to the inner silver column (0.5 mm in diameter, Length 3 mm). The average energy is 27–32 keV, the half-life is 59.6 days, and the effective radiation radius is 1.7 cm. The ^125^I seeds continuously emit low-energy γ-rays.

**Fig. 1:**
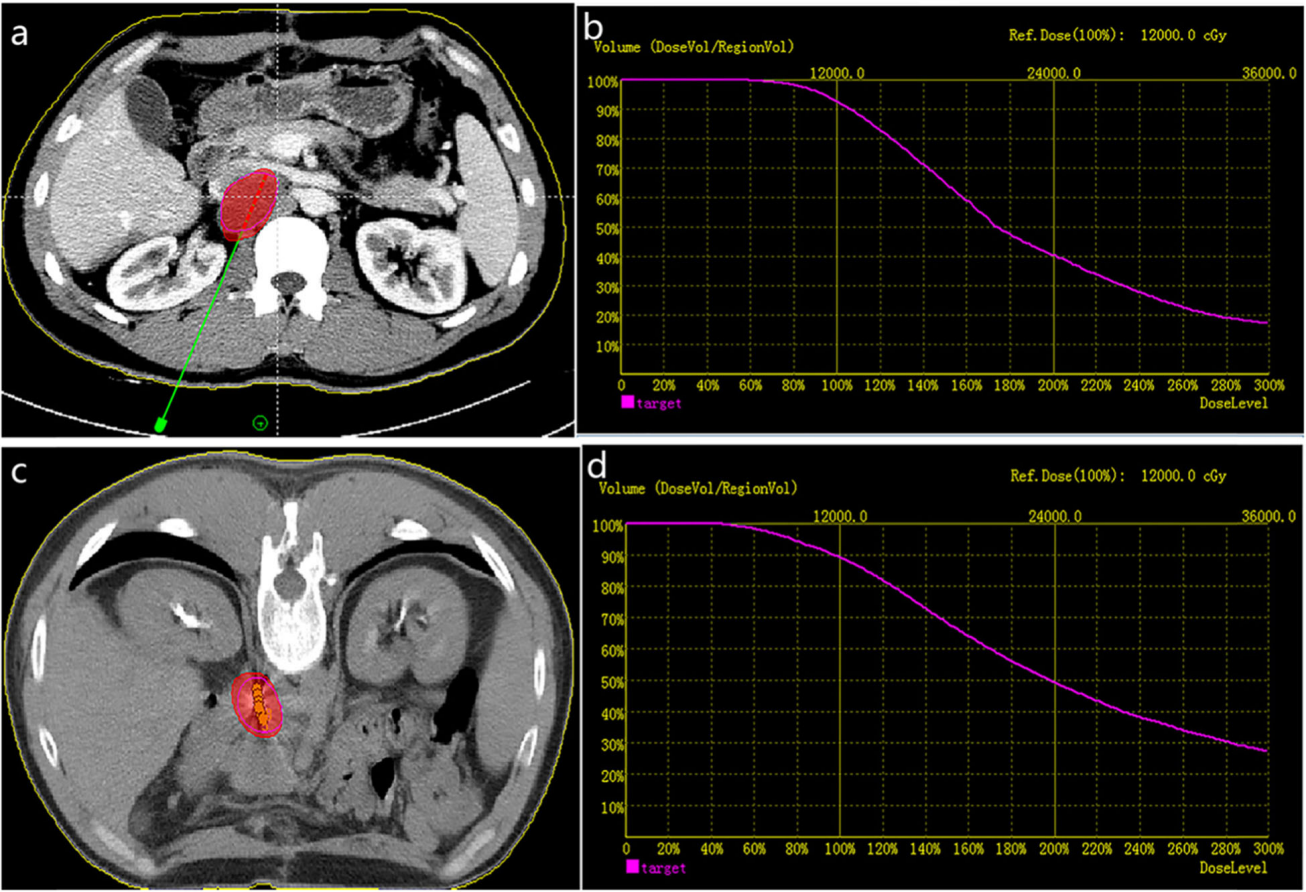
**a** Preoperative TPS, red lines represent the tumor’s contour. **b** Preoperative dose volume histograms (DVH), target = tumor. The prescribed dose (PD) was 120 Gy. A total of 90% of the tumor target (D90 = 127.5 Gy) received 127.5 Gy, and 92.5% of the tumor target received 100% of the prescribed dose (V100 = 92.5%). **c** postoperative practical distribution of seeds. **d** Postoperative DVH, D90 = 117.0 Gy, V100 = 90.0%. The postoperative dose distribution coincided roughly with preoperative distribution

Before ^125^I brachytherapy, the radiologist and physicist confirmed the clinical target volume (CTV) and the planned target volume (PTV) based on preoperative imaging (CT or MRI). As shown in Fig. 1, the required amount of ^125^I seeds, activity, and total radiation dose were calculated by the treatment planning system (TPS) (RT-RSI, Beijing Atom and High Technique Industries Inc., Beijing, China) so that D90 > matched peripheral dose. A dose-volume histogram (DVH) was then generated, the dose distribution was observed, and the seeds distribution was adjusted to achieve the optimal dose distribution in PTV. The dose within PTV should achieve 95% of the prescribed dose (Vl00 > 95%). According to our previous research experience, the prescription dose was 120 (110–140) Gy [15, 19, 21]. Fused lymph nodes means multiple lymph nodes (2–5) merge into one large lymph node. That the boundaries between the lymph nodes are unclear, and the fused lymph nodes can be clearly shown on the enhanced CT or MR before ^125^I brachytherapy. We use TPS to make a preoperative treatment plan considering the fused lymph node as only one tumor target. Similarly, we considered it as only one tumor target to release seeds during ^125^I brachytherapy.

The patient selected the appropriate position (usually in the prone or supine position, and in a few cases was also in the lateral position). According to the preoperative TPS, a puncture path was developed on the immediate CT scan image. After 5–10 ml of 1% lidocaine for local infiltration anesthesia, a 18G seed spinal needle (Yunke Pharmaceuticals Limited Liability Company, Chengdu, China) was inserted into the target lesion under CT guidance, and the direction of the needle was adjusted. Eventually, all of the needles were positioned to the farthest boundary of the tumor while ensuring that the distance between each needle was approximately 1 cm. The needle core was pulled out and the ^125^I seeds were implanted into the tumor using a ^125^I seeds implantation gun (Yunke Pharmaceuticals Limited Liability Company, Chengdu, China). Each seed was released with a distance of 0.5 cm. A final CT image was entered into TPS for postoperative dose verification.

### Follow-up and evaluation criteria

The follow-up time was defined as the interval from patient admission to death or loss of follow-up. The primary endpoint was local tumor progression-free survival (LTPFS) based on Response Evaluation Criteria in Solid Tumors (RECIST), defined as the patients with Complete response (CR), Partial response (PR), and Stable disease (SD). We only evaluated lesions for ^125^I brachytherapy, except for systemic or regional metastases. The LTPFS assessment was mainly completed by two radiologists(> 10 years of experiences)and one interventional physician(> 10 years of experiences) in our center. When the Initially evaluation results were inconsistent, the three physicians reached an agreement after consultation. The secondary study endpoint was whether CR occurred at 6 months after ^125^I brachytherapy.

### Statistical analysis

Statistical analysis was performed using SPSS 20.0 (IBM, Chicago). All of the statistical tests were bilateral, and significant differences were considered at *p* < 0.05. Pearsonχ2 and Logistic regression were used to compare qualitative data. Kaplan-Meier analysis, log-rank, and Breslow tests were used to compare LTPFS differences between different subgroups. A stratified Cox proportional hazard regression model was established and a forward stepwise method was used to incorporate the study variables to detect potential independent factors associated with LTPFS. The covariates finally included in the study were: gender, age, primary tumor, other metastasis, abnormal tumor markers, maximum diameter, the number, previous chemotherapy, previous radiotherapy, distant metastasis after ^125^I brachytherapy, D90, uniform density, necrosis, regular morphology, fusion, clear boundaries, invasion of vessels visible in image, significant enhancement, location, number of adjacent organs at risk, patients’ position, puncture path.

## Result

### Patient data

A total of 92 patients were included in the study. Patient characteristics are presented in Table 1. The preoperative average prescription dose D90 was 135.39 (112.46–162.81) Gy, and the mean V100 was 94.25% (85.6–99.9%). The average postoperative prescribed dose D90 was 144.24 (41.79–252.48) Gy, and the average V100 was 92.0% (61.4–100%). The activity was 0.8 mCi, the median operation time was 75 (30–165) minutes. For all patients, the median number of seeds was 28 (6–120). For maximum tumor diameter ≤ 3 cm, the median number of seeds was 20 (6–60). For maximum tumor diameter with 3–5 cm, the median number of seeds was 30 (12–80). For maximum tumor diameter ≥ 5 cm, the median number of seeds was 68.5 (27–120). The median postoperative hospital stay was 3 (1–24) days. The median hospitalization cost was 23,827.15 (8425.67–63,912.59) yuan. After ^125^I brachytherapy, 56 (60.9%) patients developed distant metastases at new sites.

**Table 1:** Patients’ characteristics

Gender		
Male	56	60.9%
Female	36	39.1%
Age	52.8 ± 11.0	
<60	69	75.0%
≥ 60	23	25.0%
Other parts of metastasis^a^		
Y	58	63.0%
N	34	37.0%
Abnormal tumor markers		
Y	47	51.1%
N	45	48.9%
Number		
1	67	72.8%
2	9	9.8%
≥ 3	16	17.4%
Maximum diameter (cm)	33.2 ± 17.1	
≤ 3	48	52.2%
3–5	28	30.4%
≥ 5	16	17.4%
Location		
Anterior renal vein	43	46.7%
Posterior renal vein	49	53.3%
Previous chemotherapy	8.6 ± 6.3	
<10	38	76.0%
≥ 10	12	24.0%
Previous radiotherapy^b^		
Y	8	8.7%
N	84	91.3%
Primary tumor		
Hepatic cancer	33	35.9%
Ovarian cancer	12	13.0%
Cervical cancer	8	8.7%
Colorectal cancer	10	10.9%
Others^c^	29	31.5%
NPC^d^	4	4.3%
Duodenal cancer	3	3.2%
Endometrial cancer	3	3.2%
Esophageal cancer	3	3.2%
Gastric cancer	3	3.2%
Renal cancer	2	2.2%
Pancreatic cancer	2	2.2%
Ampullary carcinoma	2	2.2%
Bladder Cancer	2	2.2%
Ureteral cancer	1	1.1%
Renal pelvic carcinoma	1	1.1%
Lung cancer	1	1.1%
Testicular cancer	1	1.1%
Embryo cancer	1	1.1%

^a^Refers to metastasis with other sites in addition to retroperitoneal lymph nodes before the treatment of ^125^I brachytherapy; ^b^8 patients received previous radiotherapy for retroperitoneal metastatic lymph nodes, 6 patients received Intensity-modulated Radiation Therapy, and 2 patients received Three Dimensional Conformal Radiation Therapy; ^c^Including renal cancer, esophageal cancer, nasopharyngeal cancer, gastric cancer, pancreatic cancer, duodenal cancer, ampullary carcinoma, endometrial cancer, bladder cancer, testicular cancer, ureteral cancer, renal pelvic carcinoma, lung cancer, embryo cancer; ^d^nasopharyngeal cancer

### Local control and complete remission

**Fig. 2:**
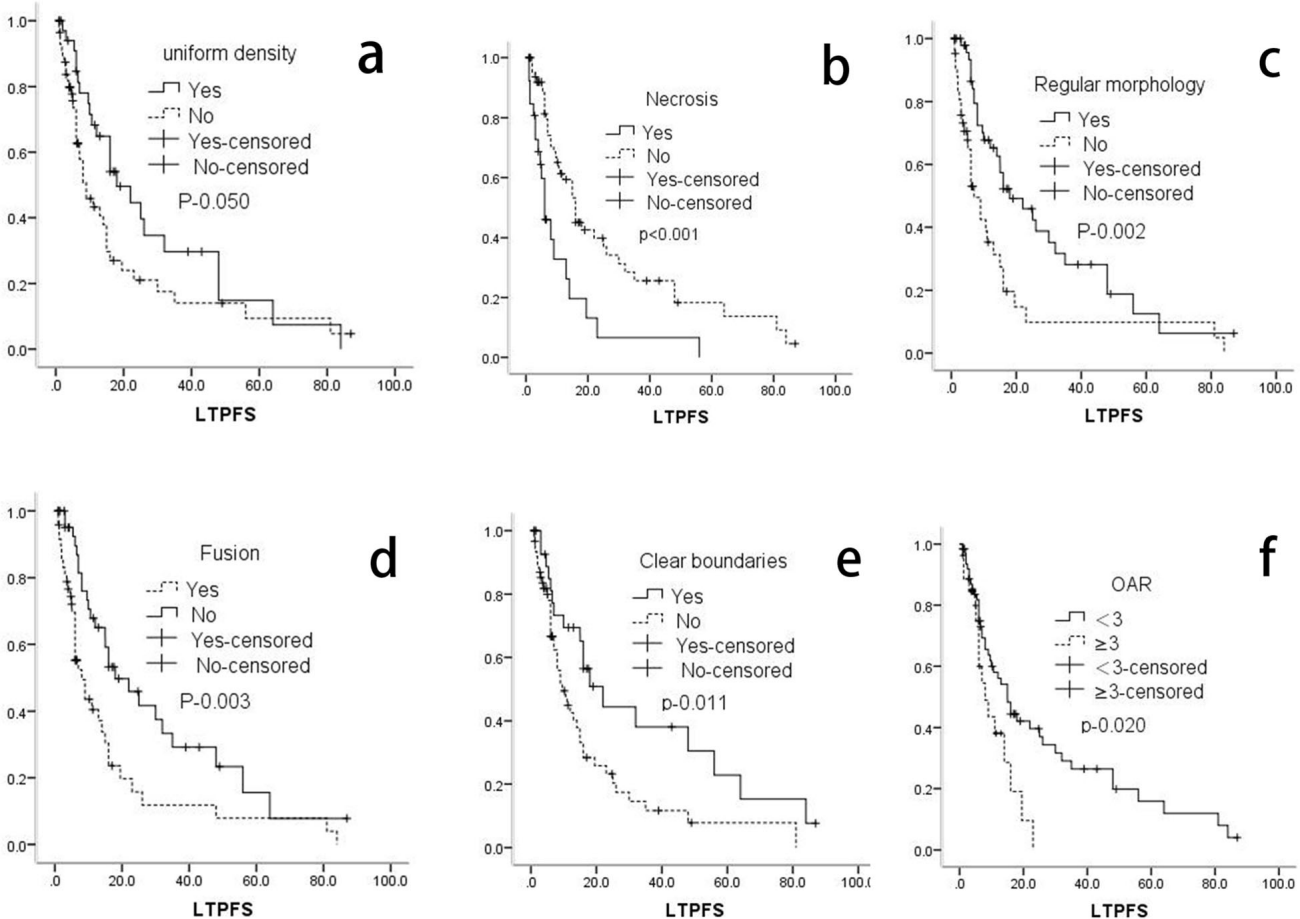
**a**-**f** represents variables, **a**. Uniform density; **b**. Necrosis; **c**. Regular morphology; **d**. Fusion; **e**. Clear boundary; **f**. OAR, The organs at risk

The local control rates at 3, 6, 12, 24, and 36 months were 89.1, 56.5, 39.1, 18.5, and 10.9%, respectively. There were 27 patients (29.3%) who achieved CR at 6 months after brachytherapy (Fig. 2 showed a patient with CR). As shown in Table 2, patients with lymph nodes that showed uniform density (OR 2.407; 95% CI 1.497, 3.870; *P* < 0.001) and clear boundaries (OR 2.751; 95% CI 1.572, 4.875; *p* < 0.001) had a higher rate of CR. Patients with ovarian cancer (OR 0.159; 95% CI 0.037, 0.693; P-0.014), a maximum tumor diameter ≤ 3 cm (OR 0.067; 95% CI 0.008, 0.545, p-0.012), or regular morphology (OR 2.051; 95% CI 1.477, 2.848; *p* < 0.001) experienced longer CR. Lymph nodes with anterior renal vein also fared better (OR 0.361; 95% CI 0.138, 0.942; p-0.034). Inferior CRs were associated with necrotic lymph nodes (OR 0.201; 95% CI 0.051, 0.790; p-0.004), fusion (OR 0.411; 95% CI 0.212, 0.799; p-0.001), invasion of vessels visible on images (OR 0.241; 95% CI 0.095, 0.607; *p* < 0.001), and significant enhancement (OR 0.616; 95% CI 0.393, 0.966; p-0.013).

**Table 2:** Univariate analysis of CR

	OR	95%CI	*P*
Gender (Male)	0.963	0.667,1.390	0.838
Age(<60)	0.915	0.692,1.209	0.509
Primary tumor^a^			**0.025**
Hepatic cance	1.182	0.359,5.581	0.783
Ovarian cancer	0.159	0.037,0.693	0.014
Cervical cancer	2.227	0.232,2.373	0.488
Colorectal cancer	0.477	0.104,2.192	0.342
Others^*^			
Other metastases^*^	1.250	0.487,3.211	0.643
Abnormal tumor Markers	1.494	1.022,2.185	0.054
Number^b^			0.075
1	0.336	0.070,1.616	0.173
2	0.114	0.016,0.828	0.032
3			
Maximum diameter^c^			**<0.001**
≤ 3 cm	0.067	0.008,0.545	0.012
3-5 cm	0.867	0.072,10.382	0.910
≥ 5 cm			
Chemo ≥10*	1.270	0.471,3.472	0.639
Previous Radio*	1.444	0.371,5.623	0.596
Postoperative metastasis	1.337	0.979,1.827	0.094
D90 ≥ 120Gy^#^	1.108	0.629,1.648	0.943
Uniform density^#^	2.407	1.497,3.870	**<0.001**
Necrosis^#^	0.201	0.051,0.790	**0.004**
Regular morphology ^#^	2.051	1.477,2.848	**<0.001**
Fusion^#^	0.411	0.212,0.799	**0.001**
Clear boundaries^#^	2.751	1.572,4.875	**<0.001**
Invasion of vessels visible on image^#^	0.241	0.095,0.607	**<0.001**
Significant enhancement^#^	0.616	0.393,0.966	**0.013**
Location^d^			
Anterior^#^ renal vein	0.361	0.138,0.942	**0.034**
Posterior renal vein^#^			
OAR<3*	1.221	0.945,1.577	0.168
Patients’ position^e^			0.772
Supine	1.500	0.119,18.836	0.753
Prone	1.056	0.090,12.418	0.966
Lateral position			
Puncture path^f^			0.284
Hepatic approach	2.586	0.767,8.590	0.126
Abdominal wall	1.027	0.274,3.851	0.968
Paravertebral approach			

^a, b, c, d, e, f^the last variable as a reference; Others * Including renal cancer, esophageal cancer, nasopharyngeal cancer, gastric cancer, pancreatic cancer, duodenal cancer, ampullary carcinoma, endometrial cancer, bladder cancer, testicular cancer, ureteral cancer, renal pelvic carcinoma, lung cancer, embryo cancer; other metastases *, Refers to metastasis with other sites in addition to retroperitoneal lymph nodes before the treatment of ^125^I brachytherapy; Chemo *, Chemotherapy; Radio *, Radiotherapy; *OAR, The organs at risk; ^#^ The assessment of image feature was mainly completed by two radiologists(> 10 years of experiences in imaging diagnosis) in our center. When the Initially evaluation results were inconsistent, need to reach an agreement after negotiation

### Univariate analysis of LTPFS

**Fig. 3:**
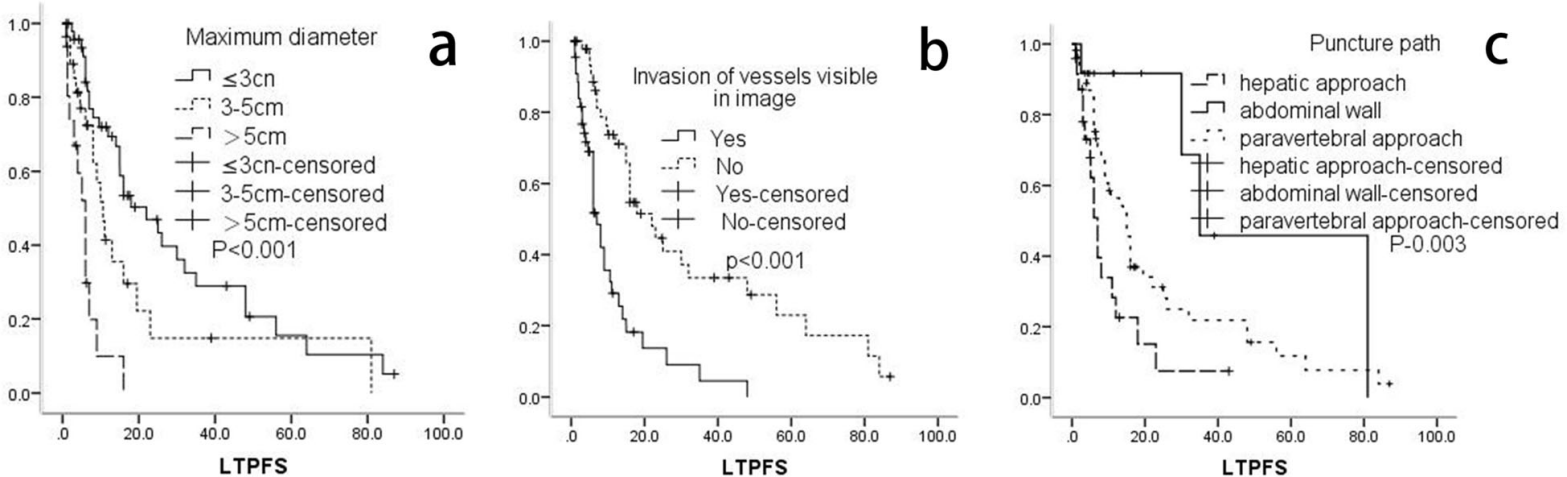
**a**. Maximum diameter; **b**. Invasion of vessels visible on image; **c**. Puncture path

The median LTPFS was 8 (1–87) months and the average LTPFS was 15.2 months. As shown in Table 3 and Fig. 3: Patients with previous radiotherapy (p-0.017), uniform density (p-0.009), clear boundary (p-0.011), regular morphology (*P* < 0.001), and organs at risk (OAR) < 3 (p-0.020) achieved better LTPFS. There was worse LTPFS in patients where lymph nodes showed necrosis (*p* < 0.001), fusion (p-0.003), and invasion of vessels visible on scans (*p* < 0.001). A puncture path with paravertebral approach through the abdominal wall was associated with better LTPFS (*P* < 0.05). The maximum diameter of the lymph nodes (P-0.042) was also related to LTPFS. A maximum diameter ≤ 3 cm (≤3 cm/≥5 cm: P-0.031), or 3–5 cm (3–5 cm/≥5 cm: P-0.018) was also significantly associated with better LTPFS than maximum diameter ≥ 5 cm.

**Table 3:** Univariate analysis of LTPFS

	*P*
Gender (Male)	0.137
Age(<60)	0.418
Primary tumor^a^	0.069
Hepatic cance	0.217
Ovarian cancer	0.003
Cervical cancer	0.845
Colorectal cancer	0.962
Others*	
Other metastases^*^	0.140
Abnormal tumor Markers	0.636
Number^b^	0.520
1	0.758
2	0.147
≥ 3	
Maximum diameter^c^	**<0.001**
≤ 3 cm	<0.001
3-5 cm	0.004
≥ 5 cm	
Chemo ≥10*	0.620
Previous Radio*	**0.017**
Postoperative metastasis	0.999
D90 ≥ 120Gy	0.687
Uniform density^#^	**0.009**
Necrosis^#^	**<0.001**
Regular morphology ^#^	**0.002**
Fusion^#^	**0.003**
Clear boundaries^#^	**0.011**
Invasion of vessels visible on image^#^	**<0.001**
Significant enhancement^#^	0.107
Location^d^	0.098
Anterior renal vein^#^	
Posterior renal vein^#^	
OAR<3*	**0.020**
Patients’ position^e^	0.766
Supine	0.433
Prone	0.534
Lateral position	
Puncture path^f^	**0.003**
Hepatic approach	0.014
Abdominal wall	0.072
Paravertebral approach	

^a, b, c, d, e f^the last variable as a reference; Others * Including renal cancer, esophageal cancer, nasopharyngeal cancer, gastric cancer, pancreatic cancer, duodenal cancer, ampullary carcinoma, endometrial cancer, bladder cancer, testicular cancer, ureteral cancer, renal pelvic carcinoma, lung cancer, embryo cancer; other metastases *, Refers to metastasis with other sites in addition to retroperitoneal lymph nodes before the treatment of ^125^I brachytherapy; Chemo *, Chemotherapy; Radio *, Radiotherapy; *OAR, The organs at risk; ^#^ The assessment of image feature was mainly completed by two radiologists(> 10 years of experiences in imaging diagnosis) in our center. When the Initially evaluation results were inconsistent, need to reach an agreement after negotiation

### Multi-factor analysis of LTPFS

**Fig. 4:**
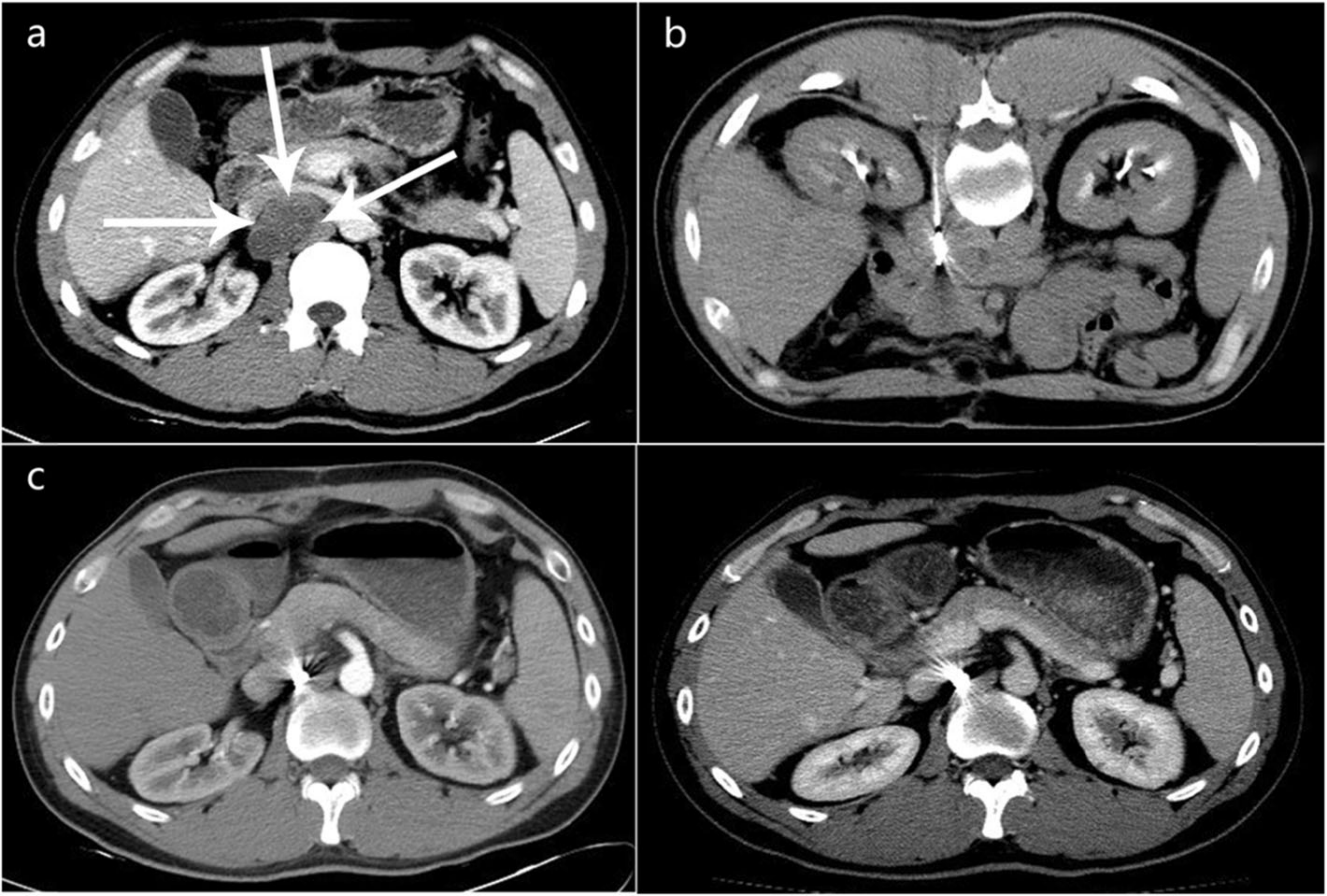
A 34-year-old male patient with retroperitoneal metastatic lymph nodes from primary hepato cellular carcinoma. **a**. Preoperative enhanced CT showed metastatic lymph nodes (arrow) with size of 42mm * 21mm, adjacent to the left renal vein, inferior vena cava, and abdominal aorta. **b**. Intraoperative CT scan. **c**, **d**. 4months after ^125^I brachytherapy, the lesion had disappeared.

As shown in Table 4 and Fig. 4, the Cox proportional hazard model suggests that the maximum diameter of the lymph nodes, invasion of vessels visible on images, and the puncture path were independent factors for LTPFS (*p* < 0.05). Multivariate analysis showed that a smaller diameter was a protective factor for LTPFS (P-0.042). Patients with lymph nodes with maximum diameters < 3 cm had significantly longer LTPFS (HR 0.252; 95% CI 0.072, 0.883; P-0.031) in comparison to maximum diameters ≥5 cm. Longer LTPFS was also observed in lymph nodes with maximum diameters of 3–5 cm (HR 0.349; 95% CI 0.146, 0.835; P-0.018), in comparison to maximum diameters ≥5 cm. Lymph nodes with invasion of vessels visible on images were also an independent risk factor for LTPFS (HR 0.380; 95% CI 0.168, 0.862; P-0.021). The puncture path was also an independent factor affecting LTPFS (p-0.007). Puncture path through the abdominal wall was associated with better LTPFS (HR 2.584; 95% CI 1.256, 5.312; P-0.010) than a hepatic approach. Additionally, there was no difference in LTPFS between an abdominal wall approach and a paravertebral approach (HR 0.410; 95% CI 0.131, 1.285; P-0.126). Kaplan-Meier (log-rank test) analysis also showed no statistical difference between the two paths (P-0.072).

**Table 4:** Multivariate analysis of LTPFS

	HR	95%CI	*P*
Maximum diameter^a^			**0.042**
≤ 3 cm	0.252	0.072,0.883	0.031
3-5 cm	0.349	0.146,0.835	0.018
≥ 5 cm			
Previous Radio	2.337	0.731,7.468	0.152
Uniform density^#^	0.756	0.359.1.593	0.462
Regular morphology ^#^	1.553	0.635,3.794	0.335
Necrosis^#^	0.604	0.284,1.286	0.191
Fusion^#^	1.555	0.659,3.688	0.313
Clear boundaries^#^	1.330	0.541,3.270	0.534
Invasion of vessels visible on image^#^	0.380	0.168,0.862	**0.021**
OAR<3	0.524	0.226,1.213	0.131
Puncture path^b^			**0.007**
Hepatic approach	2.584	1.256,5.312	0.010
Abdominal wall	0.410	0.131,1.285	0.126
Paravertebral approach			

^a, b^the last variable as a reference. # The assessment of image feature was mainly completed by two radiologists(> 10 years of experiences in imaging diagnosis) in our center. When the Initially evaluation results were inconsistent, need to reach an agreement after negotiation

### Complication

As shown in Table 5, none of the patients died directly from severe complications associated with ^125^I brachytherapy. The most common complication was pain (25%), and was alleviated by oral or injectable painkillers. One patient also had a rare needle-track metastasis.

**Table 5:** Complication

	n	%
Pain	23	25.0%
Bleeding	3	3.3%
Seed migration	1	1.1%
Needle metastasis	1	1.1%
Retroperitoneal hematoma	1	1.1%

## Discussion

Retroperitoneal lymph node metastasis occurs in most pelvic and abdominal malignant tumors at different stages of the primary disease. Due to its unique anatomical location, some primary tumors are controlled after surgery or radiotherapy or chemotherapy. However, retroperitoneal metastatic lymph nodes become a difficult problem to treat [22–25]. These lymph nodes can contribute to metastatic spread, thereby affecting the long-term survival of patients [4, 5, 26–30].

^125^I brachytherapy, with a higher local concentration of radiotherapy, is particularly well suited for the treatment of retroperitoneal metastasis [31–34]. Yao [19] et al. reported 17 patients with 19 retroperitoneal metastatic lymph nodes who received ^125^I brachytherapy with an overall effective rate of 100%. The local control rates at 6, 12, and 24 months were 88.0, 63.2, and 42.1%, respectively. Gao [20] et al. reported 20 cases of patients with primary hepatic cancer. The local control rates at 3, 6, 10, and 15 months were 70.0, 56.3, 44.4, and 25.0%, respectively. In our study, the local control rates at 3, 6, 12, 24, and 36 months were 89.1, 56.5, 39.1, 18.5, and 10.9%, respectively. The median LTPFS was 8 (1–87) months and the average LTPFS was 15.2 months. The local control rate in our study is similar to that reported by Gao et al., which is slightly lower than that reported by Yao et al. The observed differences may be due in large part to the types of primary tumors evaluated in these different studies. Our sample size was larger and contained a larger variety of primary cancers.

Previous studies don’t further explore the factors that can affect clinical efficacy and survival of ^125^I brachytherapy [15–19]. Therefore, the main purpose of our study was to explore the potential factors that could influence the clinical efficacy of ^125^I brachytherapy. There was a significant relationship between CR and factors including: density, border, morphology, necrosis, fusion, and invasion of vessels visible on scans (*p* < 0.05). These variables are all imaging features that reflect the malignant degree and invasiveness of the cancers. The final statistical analysis also confirmed that the tumor pathology was also associated with the occurrence of CR (p-0.025). When the lymph nodes were characterized by uneven density, unclear borders, irregular morphology, necrosis, fusion, and invasion of vessels visible on images, not only was the degree of malignancy high, but the tumor growth was also accelerated. At the same time, when performing ^125^I brachytherapy, these imaging features make it difficult to determine the true extent of invasion, which not only affects the accuracy of the preoperative treatment planning, but also affects the arrangement of the intraoperative seeds. These limitations can result in incomplete target coverage, thereby making it difficult to obtain CR. Retroperitoneal lymph nodes are located near the renal vein and are surrounded by a rich network of blood vessels. Therefore, there is a high likelihood that these lymph nodes will invade large blood vessels. Additionally, with more OARs, the technical execution of brachytherapy is increasingly more difficult. Therefore, location of lymph nodes is crucial factors that can affect clinical efficacy.

One of the main advantages of ^125^I brachytherapy is the high local control [16, 21, 35–37]. Therefore, we used LTPFS as the primary end point, and used Kaplan-Meier analysis and log-rank test to explore the factors affecting LTPFS. The final statistical analysis showed that invasion of vessels visible on images, borders, fusion, necrosis, density, and morphology all influenced LTPFS. These imaging features can affect the accuracy of the preoperative treatment plan and the arrangement of the intraoperative seeds. Therefore, it is also a related factor of LTPFS. Puncture path and OAR are also factors that influenced LTPFS. This is related to the technical factors of ^125^I brachytherapy. For target lesions associated with more OARs, the planned dose and arrangement of the intraoperative seeds were decreased, resulting in insufficient dose and coverage of the target. Univariate analysis and multivariate analysis indicated that the puncture path was an independent factor affecting LTPFS, as there was better LTPFS associated with an approach through the abdominal wall and paravertebral space. In the case of other approaches, a puncture path through the liver and intestine can considered to be a secondary option. The lymph nodes through the liver puncture are usually located close to the biliary tract, gastrointestinal tract, and blood vessels, making the operation more complex. The precise distribution of seeds is difficult to guarantee, so the outcome is typically inferior LTPFS.

Previous studies have shown that the maximum diameter is an independent factor affecting the LTPFS of ^125^I brachytherapy [21]. The same conclusion was obtained in this study. The maximum diameter was not only related to CR, but it was also an independent factor affecting LTPFS. Another finding in this study was that metastatic lymph nodes that invaded the vessels visible on scans were an independent risk factor for LTPFS (HR 0.380; 95% CI 0.168, 0.862; P-0.021). Careful preoperative planning and intraoperative execution were essential in order to avoid damaging large blood vessels and causing hemorrhaging. This leads to an increase in the degree of difficulty of the operation. At the same time, it is difficult to distinguish the vessels on CT during the operation, which leads to possible deviations from the preoperative plan. Consequently, there may be incomplete target coverage and inefficient radiotherapy delivered to target lesions, which eventually leads to a high recurrence and lower LTPFS.

The median overall survival (OS) was 15.45 (1–109) months, and the average OS was 23.28 months. We mainly studied the LTPFS of peritoneal metastases with ^125^I brachytherapy rather than OS, and 60.9% of patients developed distant metastases at new sites after ^125^I brachytherapy, so, we didn’t further analyze the OS.

In our study, one patient, a 50-year-old woman with cervical adenocarcinoma, had a rare needle-track metastasis. She underwent previous Intensity-modulated Radiation Therapy for retroperitoneal metastatic lymph nodes. Preoperative enhanced CT showed liquefaction necrosis inside the lymph nodes. One month after seed implantation, postoperative enhanced CT can be seen that strip-shaped soft tissue focus appears in the subcutaneous muscle along the original puncture path, but does not break through the skin. The patient refused further puncture biopsy to confirm the diagnosis, and a multidisciplinary discussion suspected needle-track metastasis. Because the patient was associated with multiple site metastases, palliative chemotherapy and supportive treatment were subsequently selected, the patients’ OS was 13.1 months. The incidence of needle-track metastasis in percutaneous lung biopsy is 0.012% [38]. Few studies reported needle-track metastasis after ^125^I brachytherapy for cancers [39], our study was 1.1%. The possible reason is that tumor cells are more likely to flow along the puncture path and diffuse and metastasize in the liquefied necrotic lymph nodes.

Our study is limited by its retrospective nature. The retroperitoneal anatomy is complex, the intraoperative puncture path and seed arrangement were susceptible to the operator’s technique, making it difficult to accurately perform preoperative planning. In addition, it is difficult to implement a controlled study compared with radiotherapy due to the large number of primary tumor types. Based on good results, we will consider a comparative study design for a single primary in the next.

## Conclusion

CT-guided ^125^I brachytherapy is an optional palliative treatment modality for retroperitoneal metastatic lymph nodes, which can provide high local control without severe complications. Its clinical efficacy is not only related to the lymph node itself, but also related to the technology of ^125^I brachytherapy. Better preoperative planning, intraoperative implementation, better choice of puncture path, and selection of appropriate tumor size are crucial considerations for the improvement of the clinical efficacy of ^125^I brachytherapy for retroperitoneal metastatic lymph nodes.

## Data Availability

This submission has been successfully deposited into the Research Data Deposit with the number:(RDD) RDDA2017000140.

## Abbreviations

CI: Confidence interval
CR: Complete response
CT: Computed tomography
CTV: Clinical target volume
DNA: Deoxyribose Nucleic Acid
DVH: Dose volume histogram
ECOG: Eastern Cooperative Oncology Group
HR: Hazard ratio
LTPFS: Local tumor free progression survival
MRI: Magnetic resonance imaging
OAR: Organs at risk
OR: Odds ratio
PACS: Picture Archiving and Communication Systems
PD: Disease progression
PET: Positron emission tomography
PR: Partial response
PTV: Planning target volume
RECIST: Response Evaluation Criteria in Solid Tumors
SD: Stable disease
TPS: Treatment planning system
